# The association between *NAT2* acetylator status and adverse drug reactions of sulfasalazine: a systematic review and meta-analysis

**DOI:** 10.1038/s41598-020-60467-8

**Published:** 2020-02-27

**Authors:** Jeong Yee, So Min Kim, Ji Min Han, Nari Lee, Ha Young Yoon, Hye Sun Gwak

**Affiliations:** 1 Division of Life and Pharmaceutical Sciences, Ewha Womans University, 52 Ewhayeodae-gil, Seodaemun-gu Seoul, 03760 Republic of Korea; 2 College of Pharmacy, Ewha Womans University, 52 Ewhayeodae-gil, Seodaemun-gu Seoul, 03760 Republic of Korea; 3 Graduate School of Pharmaceutical Sciences, Ewha Womans University, 52 Ewhayeodae-gil, Seodaemun-gu Seoul, 03760 Republic of Korea

**Keywords:** Genetics research, Risk factors

## Abstract

*N*-acetyltransferase 2 (NAT2) acetylator status can be classified into three groups depending on the number of rapid alleles (e.g., NAT2*4): rapid, intermediate, and slow acetylators. Such acetylator status may influence the occurrence of adverse drug reactions (ADRs) during sulfasalazine treatment. This systematic review and meta-analysis aimed to evaluate the association between *NAT2* acetylator status and ADRs of sulfasalazine. We searched for qualified studies in PubMed, Web of Science, Embase, and the Cochrane Library. Odds ratio (OR) and 95% confidence intervals (CIs) were calculated to evaluate the strength of the association between *NAT2* acetylator status and ADRs of sulfasalazine. Nine cohort studies involving 1,077 patients were included in the meta-analysis. *NAT2* slow acetylators were associated with an increase in overall ADRs (OR 3.37, 95% CI: 1.43 to 7.93; *p* = 0.005), discontinuation due to overall ADRs (OR 2.89, 95% CI: 1.72 to 4.86; *p* < 0.0001), and dose-related ADRs (OR 5.20, 95% CI: 2.44 to 11.08; *p* < 0.0001), compared with rapid and intermediate acetylators. In conclusion, *NAT2* slow acetylators are at risk of ADRs during sulfasalazine treatment. Based on our findings, *NAT2* genotyping may be useful to predict the occurrence of ADRs during sulfasalazine treatment.

## Introduction

Sulfasalazine is one of the classical agents used to treat rheumatoid arthritis^1^. It has also been widely used to treat other autoimmune diseases, such as ankylosing spondylitis, Crohn’s disease, and ulcerative colitis^2–4^. Although the mechanism of sulfasalazine action is not well established, the drug is known to have anti-inflammatory and immunomodulatory effects^1^.

The common adverse drug reactions (ADRs) of sulfasalazine are gastrointestinal symptoms (including nausea, vomiting, dyspepsia, and anorexia), headache, dizziness, and rash^1,5^. Severe or fatal ADRs such as hematologic disorders (including leukopenia), systemic hypersensitivity reactions, lupus-like syndromes, hepatotoxicity, and pulmonary complications can occur, even though the incidence is low^1,6^. Most ADRs generally occur within the first few months of starting sulfasalazine treatment, and about 20–30% of patients discontinue the drug during this period because of ADRs^7,8^. Thus, the occurrence of ADRs is an important factor influencing sulfasalazine treatment continuation.

Sulfasalazine consists of two components, 5-aminosalicylate (5-ASA) and sulfapyridine (SP), which are connected by an azo bond^9^. After oral administration, approximately 15–30% of sulfasalazine is absorbed in the small intestine^10,11^, and the rest is metabolized in the colon to 5-ASA and SP by bacterial azoreductase^10,12,13^. About 25% of 5-ASA is absorbed as the unchanged form. SP, however, which is highly associated with sulfasalazine ADRs, is mostly absorbed from the colon, acetylated by *N*-acetyltransferase 2 (NAT2) in the liver, and then eliminated renally^10,12^.

The *NAT2* gene, located on human chromosome 8, is highly polymorphic^14^. Although allele frequencies of the *NAT2* gene differ widely across ethnicities^15^, NAT2*4 is regarded as the wild-type allele^16^. While NAT2*4, which is considered a rapid allele, maintains NAT2 activity, several mutated alleles that reduce enzyme activity have been identified (e.g., NAT2*5, NAT2*6, NAT2*7); these alleles are considered slow alleles^17^. As *NAT2* shows excellent genotype–phenotype correlations, *NAT2* genotypes can be classified into three phenotypes: rapid acetylators (RAs; carrying two rapid alleles), intermediate acetylators (IAs; one rapid allele and one slow allele) and slow acetylators (SAs; two slow alleles)^18^. Or, the genotypes can be categorized into two groups depending on whether at least one NAT2*4 allele is present or not.

Since NAT2 plays an important role in sulfasalazine metabolism, several previous studies assessed the association between *NAT2* acetylator status and sulfasalazine ADRs. However, study results were inconsistent, potentially because of different ethnicities and disease populations. Also, the individual studies had limited statistical power because of relatively small sample sizes. Therefore, we performed a systematic review and meta-analysis to determine the association between *NAT2* acetylator status and sulfasalazine ADRs.

## Results

### Identification and characteristics of the included studies

**Figure 1:**
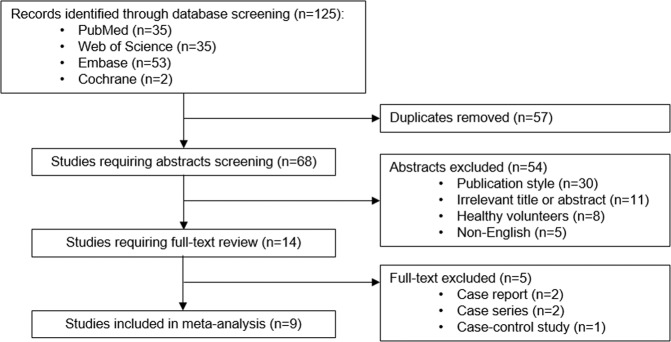
Flow diagram of study selection.

The study selection process is shown in Fig. 1. A total of 125 records were identified from searches of four databases. After removing 57 duplicates, 68 studies remained. Among them, 54 studies were removed during title and abstract screening, and 14 were selected for full-text review. Five studies were excluded during full-text review because of the study design: two case reports^19,20^, two case series^21,22^, and one case-control study^23^. Finally, nine studies^24–32^ with 1,077 patients were included for meta-analysis.

The main characteristics of each study are summarized in Table 1. The studies were conducted mainly in Asian populations, although one study was performed in Caucasians, and two studies included mixed populations (mostly Caucasians). Participants’ diseases varied among individual studies. Although studies examined different single nucleotide polymorphism (SNPs), they selected some of the seven SNPs known as important polymorphisms that cause reduced enzyme activity^33^. Among five studies in which the Hardy-Weinberg equilibrium (HWE) test was performed^25,26,29,31,32^, two studies were not in HWE^25,32^. Quality scores evaluated by the Newcastle-Ottawa Quality Assessment Scale (NOS) ranged from 4 to 8.

**Table 1:** Characteristics of studies included in the meta-analysis.

Study (year)	Sample size	Ethnicity	Study design	NOS score	Disease	Sulfasalazine dose	Genotyping method	SNPs for genotyping
Sabbagh *et al*.^24^	11	Mixed	Prospective cohort study	6	CDLE	0.5–2 g/day	PCR-RFLP or allele-specific PCR	rs1208 rs1041983 rs1799929 rs1799930 rs1799931 rs1801279 rs1801280
Ricart *et al*.^25^	64	Caucasian	Retrospective cohort study	6	UC	1–4 g/day	DNA microarray or DNA sequencing	rs1208 rs1041983 rs1799929 rs1799930 rs1799931 rs1801279 rs1801280
Tanaka *et al*.^26^	144	Japanese	Retrospective cohort study	6	RA	0.5–1.5 g/day	PCR-RFLP or allele-specific PCR	rs1208 rs1041983 rs1799929 rs1799930 rs1799931 rs1801279 rs1801280
Tanigawara *et al*.^27^	13	Japanese	Retrospective cohort study	4	IBD	0.5–6 g/day	PCR-RFLP	rs1799929 rs1799930 rs1799931
Kumagai *et al*.^28^	96	Japanese	Retrospective cohort study	5	RA	0.5–1 g/day	PCR-RFLP	rs1799929 rs1799930 rs1799931
Chen *et al*.^29^	68	Han Chinese	Prospective cohort study	6	IBD	NA	PCR-RFLP	rs1799929 rs1799930 rs1799931
Taniguchi *et al*.^30^	186	Japanese	Retrospective cohort study	4	RA	0.5–1.5 g/day	TaqMan	rs1041983 rs1799929 rs1799930 rs1799931
Hou *et al*.^31^	266	Han Chinese	Prospective cohort study	8	AS	1.5–3 g/day	PCR-RFLP	rs1799929 rs1799930 rs1799931
Wiese *et al*.^32^	229	Mixed	Prospective cohort study	8	RA	0.5–3 g/day	TaqMan	rs1041983 rs1801280

AS: ankylosing spondylitis; CDLE: chronic discoid lupus erythematosus; IBD: inflammatory bowel disease; NA: not available; NOS: Newcastle‐Ottawa Scale; PCR: polymerase chain reaction; RA: rheumatoid arthritis; RFLP: restriction fragment length polymorphism; SNP: single nucleotide polymorphism; UC: ulcerative colitis.

### Quantitative data synthesis

**Figure 2:**
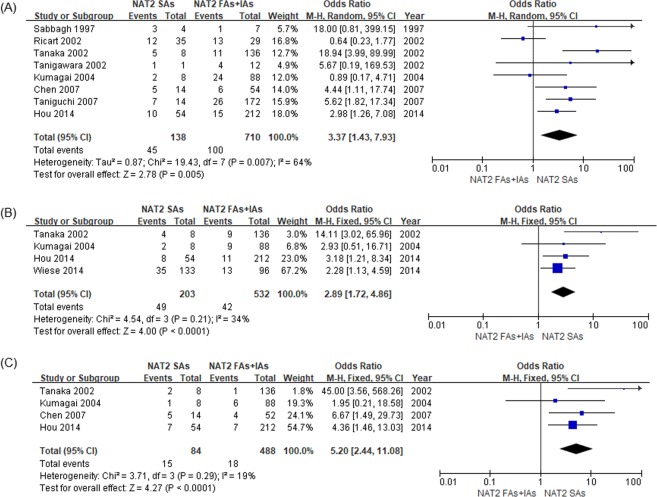
Forest plots of the association between *NAT2* acetylator status and adverse drug reactions (ADRs) of sulfasalazine. (**A**) Overall ADRs. (**B**) Discontinuation due to overall ADRs. (**C**) Dose-related ADRs. RAs: rapid acetylators; IAs: intermediate acetylators; SAs: slow acetylators.

The meta-analysis results comparing sulfasalazine ADRs between *NAT2* SAs and RAs + IAs are shown in Fig. 2. Eight studies were included in meta-analysis for the primary outcome, and SAs were significantly associated with an increase in overall ADRs, compared with RAs + IAs (Odds ratio [OR] 3.37, 95% confidence interval [CI]: 1.43 to 7.93; *p* = 0.005). Since significant heterogeneity was observed (*I*^2^ = 64%, *p* = 0.007), a random-effects model was used. For secondary outcomes, SAs had significantly increased risks of discontinuation due to overall ADRs (OR 2.89, 95% CI: 1.72 to 4.86; *p* < 0.0001) and dose-related ADRs (OR 5.20, 95% CI: 2.44 to 11.08; *p* < 0.0001) compared with RAs + IAs, using a fixed-effects model (*I*^2^ = 34%, *p* = 0.21; and *I*^2^ = 19%, *p* = 0.29, respectively).

Five studies were included in comparison of the three *NAT2* acetylator statuses (Table 2). *NAT2* SAs were significantly associated with increased overall ADRs, compared with RAs or IAs (SAs vs RAs: OR 3.56, 95% CI: 1.73 to 7.35; *p* = 0.0006; SAs vs IAs: OR 4.70, 95% CI: 1.24 to 17.89; *p* = 0.02). However, there was no significant difference between IAs and RAs (OR 1.01, 95% CI: 0.57 to 1.82; *p* = 0.96).

**Table 2:** Summary of meta-analysis between *NAT2* acetylator status and overall adverse drug reactions of sulfasalazine.

*NAT2* acetylator status comparison	Number of studies	First comparator		Second comparator		*I*^2^ (%)	Statistical model	Odds ratio (95% CI)	*p*-value
		Case	Control	Case	Control				
SAs vs RAs	5	21	54	29	201	24	Fixed	3.56 (1.73–7.35)	0.0006
SAs vs IAs	5	21	54	26	199	58	Random	4.70 (1.24–17.89)	0.02
IAs vs RAs	5	26	199	29	201	0	Fixed	1.01 (0.57–1.82)	0.96

RAs: rapid acetylators; IAs: intermediate acetylators; SAs: slow acetylators.

**Figure 3:**
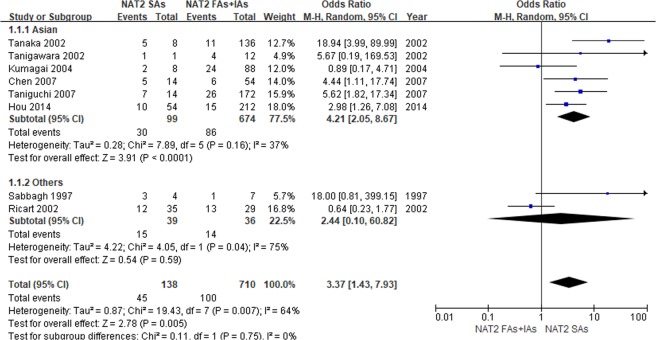
Forest plots of the association between *NAT2* acetylator status and overall adverse drug reaction of sulfasalazine when stratified by ethnicity. RAs: rapid acetylators; IAs: intermediate acetylators; SAs: slow acetylators.

Subgroup analysis by ethnicity was performed for the primary outcome (Fig. 3). When *NAT2* SAs were compared with RAs + IAs, a significant association was found between *NAT2* acetylator status and overall ADRs in Asians (OR 4.21, 95% CI: 2.05 to 8.67; *p* < 0.0001). However, there was no significant difference in other populations, possibly due to the small number of studies included.

We additionally performed subgroup analyses on studies with prospective design and rheumatoid arthritis to rule out the confounding effects due to the different study designs, diseases, and doses. Results from studies with prospective design showed that the OR of SAs compared to RAs was 2.97 (95% CI: 1.67–5.28, *p* = 0.0002). In the analysis using studies on rheumatoid arthritis, OR of SAs compared to RAs was 3.14 (95% CI: 1.07–9.24, *p* = 0.04). The subgroup analysis results were similar to that from the entire meta-analysis (OR: 3.56, 95% CI: 1.73–7.35).

### Sensitivity analysis and publication bias

**Figure 4:**
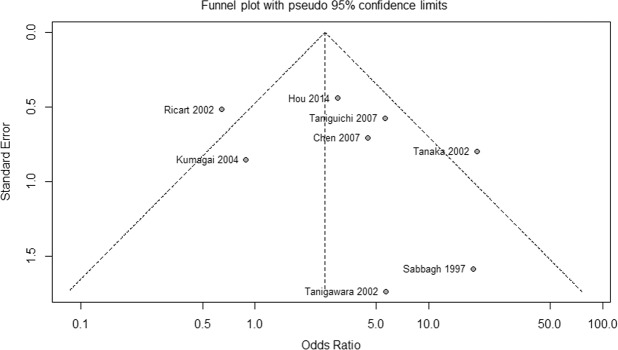
Funnel plot of the association between *NAT2* acetylator status and overall adverse drug reactions of sulfasalazine (slow acetylators vs rapid and intermediate acetylators).

Sensitivity analysis was conducted by sequentially excluding each study to assess the effects of individual studies on the overall meta-analysis estimate (Supplementary Table S1). Comparing overall ADRs between *NAT2* SAs and RAs + IAs, this analysis yielded similar results to those obtained before studies were omitted (OR range 2.55–4.05). When the Ricart *et al*. study^25^ was removed, heterogeneity was greatly reduced (*I*^2^ = 31%, *p* = 0.19). Moreover, Galbraith plot showed that the studies of Ricart *et al*.^25^ and Tanaka *et al*.^26^ were the major source of heterogeneity (Supplementary Fig. S1). When these two studies were removed, results of the meta-analysis remained significant (OR 3.38, 95% CI: 1.97 to 5.80; *p* < 0.0001), and heterogeneity was not observed (*I*^2^ = 0%, *p* = 0.46). A funnel plot for the primary outcome is shown in Fig. 4. Begg’s test and Egger’s test indicated that there was no evidence of publication bias among studies (Begg’s test: *p* = 0.6207; Egger’s test: *p* = 0.392).

## Discussion

This meta-analysis evaluated the association between *NAT2* acetylator status and sulfasalazine ADRs. Compared with *NAT2* RAs + IAs, SAs were significantly associated with increased overall ADRs during sulfasalazine treatment, and the significant association was maintained in the subgroup of Asian patients. Regarding secondary outcomes, SAs versus RAs + IAs had an increased risk of discontinuation due to overall ADRs, and a similar result was obtained for dose-related ADRs. The OR for dose-related ADRs for SAs was higher than that for overall ADRs, thus implying that pharmacokinetic properties are important in the occurrence of ADRs during sulfasalazine treatment.

The present results are consistent with those of several previous meta-analyses, which assessed the association between *NAT2* acetylator status and anti-tuberculosis drug-induced liver injury (AT-DILI)^34–37^. According to these studies, *NAT2* SAs were associated with an increased risk of AT-DILI. Moreover, it has been reported that slow acetylation is a risk factor for ADRs associated with other drugs, such as hydralazine^38^, amifampridine phosphate^39^, and co-trimoxazole^40^. These results could be explained by reduced NAT2 activity, which led to higher concentrations of toxic substances.

Autoimmune diseases, including rheumatoid arthritis, show heterogeneity in both pathophysiological and clinical aspects^41^. Autoimmune disease itself has diverse clinical manifestations and the etiology remains elusive^42,43^. In addition, the detailed mechanisms of adverse reactions of drugs used for autoimmune diseases have not been understood; however, SP, a sulfonamide metabolite of sulfasalazine, is known to be a main cause of ADRs during sulfasalazine treatment^5,6^. This moiety might cause sulfa-related toxicity including headache, nausea, and vomiting as well as several allergic reactions^44^. These ADRs seemed to be dose related and mostly reversible, being affected by hepatic acetylation^5^.

A previous study demonstrated that patients with ADRs during sulfasalazine treatment had increased serum concentrations of SP (>50 μg/mL); however, such increased concentrations were not observed for sulfasalazine, 5-ASA, and metabolites of SP^45^. As SP undergoes *N*-acetylation or glucuronidation, followed by hydroxylation, and is then eliminated renally, *N*-acetylation is the main route of metabolism^6,12,13^. A previous pharmacokinetic study reported that *NAT2* SAs had higher serum concentrations of SP than RAs + IAs^46^, suggesting that ADRs in SAs are associated with higher concentrations of SP. In addition, several *NAT2* phenotyping studies, which determined acetylator phenotype from the serum concentration ratio of free SP to total SP using Evan’s method^47^, instead of genotyping, reported that SAs experienced significantly more ADRs than RAs^48,49^. Therefore, the *N*-acetylation rate of SP is a possible key factor in the occurrence of ADRs.

Additionally, in comparisons of the three acetylator statuses, *NAT2* SAs were more likely to experience overall ADRs than RAs or IAs, whereas no significant difference was found between RAs and IAs. A previous meta-analysis showed similar results: Shi *et al*.^36^ found that IAs were not significantly associated with an increased risk of AT-DILI. The results suggested that having only one rapid allele may not be a risk factor for ADRs because this is adequate for metabolizing drugs and toxic substances.

This meta-analysis may still have some limitations that should be considered when interpreting the results. First, there was a limited number of studies, especially in non-Asian populations. However, according to a typical Cochrane review, the median number of included studies per review was 6 (interquartile range: 3–12)^50^. Although the small number of studies (e.g., less than 10) could make interpretation difficult in advanced tools such as Begg’s or Egger’s test^51^, Herbison *et al*. reported that meta-analysis with as few as four or five studies could produce robust results consistent with long-term results^52^. Second, participants’ diseases, ADR definitions, and SNPs used for genotyping varied among individual studies, thereby increasing heterogeneity. Third, some potential risk factors, such as sulfasalazine dose, treatment duration, and concomitant medications could not be adjusted due to lack of information from individual studies. Fourth, since only studies published in English were included in the meta-analysis, the possibility of publication bias could not be excluded, even though statistical tests demonstrated that there was no evidence of publication bias.

To our knowledge, this is the first systematic review and meta-analysis to evaluate the association between *NAT2* acetylator status and sulfasalazine ADRs. By combining inconsistent results from individual studies, we could draw the conclusion that *NAT2* SAs have an increased risk of ADRs during sulfasalazine treatment, especially in Asian populations. Therefore, to prevent the occurrence of ADRs, individualized sulfasalazine treatment strategies according to *NAT2* genotyping can be proposed based on our findings.

## Methods

### Literature search strategy

Two researchers independently searched four databases (PubMed, Web of Science, Embase, and the Cochrane Library) on 11 July 2019, for studies about the association between *NAT2* acetylator status and sulfasalazine ADRs. The following search terms were used: (sulfasalazin* OR sulphasalazin* OR salicylazosulfapyridin* OR salicylazosulphapyridin* OR salazosulfapyridin* OR Azulfidin* OR Salazopyrin*) AND (N-acetyltransferase 2 OR N-acetyl-transferase 2 OR N acetyltransferase 2 OR Nacetyltransferase 2 OR NAT2 OR arylamine acetyltransferase) AND (polymorph* OR variant* OR mutation* OR genotyp* OR phenotyp* OR haplotyp*). The search was not restricted by publication date. Duplicates and irrelevant studies were removed through the initial screening of titles and abstracts according to the eligibility criteria.

### Inclusion and exclusion criteria

The following criteria were used to identify eligible studies: (1) evaluating the association between *NAT2* acetylator status and ADRs in sulfasalazine-treated patients; (2) using prospective or retrospective cohort study design; (3) providing sufficient information to calculate OR and 95% CIs; and (4) being published in English. Exclusion criteria comprised: (1) conference or meeting abstracts, summaries, reviews, comments, letters, news, and editorials; (2) *in vitro* or animal studies; or (3) studies in healthy volunteers. If there were overlapping data, only the most recent and comprehensive data were included in the meta-analysis.

### Data extraction

All data were extracted independently by two researchers, and discrepancies were resolved by consensus. The following information was extracted from each study: name of the first author, publication year, ethnicity, study design, disease of patients, sulfasalazine dose, genotyping method, single nucleotide polymorphisms (SNPs) used for genotyping, and deviation from HWE. Also, the following outcome data were extracted from each study: the number of patients with or without overall ADRs (primary outcome), the number of patients who discontinued the drug due to overall ADRs, and the number of patients who experienced dose-related ADRs (secondary outcomes). Dose-related ADRs of sulfasalazine were defined based on a study by Taffet *et al*.^5^, and these reactions included nausea, vomiting, headache, malaise, hemolytic anemia, reticulocytosis, and methemoglobinemia.

### Assessment of study quality

Two researchers independently assessed the selected studies based on the NOS for cohort studies^53^. There are three categories in NOS: selection of study sample, comparability between case and control group, and outcome assessment. Each study can be assessed with a total score of 0–9. In this review, we rated one point in each item of comparability, if age and other known risk factors (such as sulfasalazine dose) were matched or adjusted in the analysis. The minimum follow-up period was 12 weeks, which was considered sufficient for outcomes to occur^7,8^.

### Statistical analysis

OR and 95% CIs were calculated by Z test to estimate the strength of the association between *NAT2* acetylator status and sulfasalazine ADRs. *NAT2* SAs (without the NAT2*4 allele) were compared with RAs + IAs for each outcome. Additionally, three comparisons were performed for overall ADRs: SAs vs RAs, SAs vs IAs, and IAs vs RAs. A *p*-value <0.05 was considered statistically significant. Heterogeneity between studies was assessed by a chi square-based Q test and an *I*^2^ test; *I*^2^ > 50% was considered to indicate significant heterogeneity. When there was no statistical evidence of heterogeneity, the fixed-effects model (Mantel-Haenszel method) was used, otherwise the random-effects model (DerSimonian-Laird method) was used to calculate pooled estimates^54,55^. If a study had no events in both comparison groups, the study was excluded from meta-analysis of that outcome. Subgroup analysis was performed according to ethnicity.

To assess the stability of the results, sensitivity analysis was performed by sequentially excluding each study, or by omitting outlier studies. Galbraith plot was used to spot outliers as potential sources of heterogeneity^56^. Publication bias was assessed using funnel plots, Begg’s test, and Egger’s test. When a *p*-value was <0.05, we considered it statistically significant for publication bias. All statistical analyses were performed using Review Manager (version 5.3; The Cochrane Collaboration, Copenhagen, Denmark) and R software (version 3.6.0; R Foundation for Statistical Computing, Vienna, Austria). The review followed Preferred Reporting Items for Systematic Reviews and Meta-analyses (PRISMA) guidelines^57^.

## Supplementary information

Supplementary Information.

## References

[1] Plosker GL, Croom KF (2005). Sulfasalazine: a review of its use in the management of rheumatoid arthritis. Drugs..

[2] Akkoc N, van der Linden S, Khan MA (2006). Ankylosing spondylitis and symptom-modifying vs disease-modifying therapy. Best. Pract. Res. Clin. Rheumatol..

[3] Lichtenstein GR (2018). ACG Clinical Guideline: Management of Crohn’s Disease in Adults. Am. J. Gastroenterol..

[4] Kornbluth A, Sachar DB (2010). Ulcerative colitis practice guidelines in adults: American College Of Gastroenterology, Practice Parameters Committee. Am. J. Gastroenterol..

[5] Taffet SL, Das KM (1983). Sulfasalazine. Adverse effects and desensitization. Dig. Dis. Sci..

[6] Rains CP, Noble S, Faulds D (1995). Sulfasalazine. A review of its pharmacological properties and therapeutic efficacy in the treatment of rheumatoid arthritis. Drugs..

[7] Amos RS (1986). Sulphasalazine for rheumatoid arthritis: toxicity in 774 patients monitored for one to 11 years. Br. Med. J..

[8] Donovan S, Hawley S, MacCarthy J, Scott DL (1990). Tolerability of enteric-coated sulphasalazine in rheumatoid arthritis: results of a co-operating clinics study. Br. J. Rheumatol..

[9] Khan AA, Piris J, Truelove SC (1977). An experiment to determine the active therapeutic moiety of sulphasalazine. Lancet..

[10] Azadkhan AK, Truelove SC, Aronson JK (1982). The disposition and metabolism of sulphasalazine (salicylazosulphapyridine) in man. Br. J. Clin. Pharmacol..

[11] Hoult JR (1986). Pharmacological and biochemical actions of sulphasalazine. Drugs..

[12] Peppercorn MA (1984). Sulfasalazine. Pharmacology, clinical use, toxicity, and related new drug development. Ann. Intern. Med..

[13] Klotz U (1985). Clinical pharmacokinetics of sulphasalazine, its metabolites and other prodrugs of 5-aminosalicylic acid. Clin. Pharmacokinet..

[14] Blum M, Grant DM, McBride W, Heim M, Meyer UA (1990). Human arylamine N-acetyltransferase genes: isolation, chromosomal localization, and functional expression. DNA Cell Biol..

[15] Mortensen HM (2011). Characterization of genetic variation and natural selection at the arylamine N-acetyltransferase genes in global human populations. Pharmacogenomics..

[16] Hein DW (2000). Molecular genetics and epidemiology of the NAT1 and NAT2 acetylation polymorphisms. Cancer Epidemiol. Biomarkers Prev..

[17] Cascorbi I, Brockmöller J, Mrozikiewicz PM, Müller A, Roots I (1999). Arylamine N-acetyltransferase activity in man. Drug. Metab. Rev..

[18] Stanley LA, Sim E (2008). Update on the pharmacogenetics of NATs: structural considerations. Pharmacogenomics..

[19] Furukawa K, Ohtani T, Furukawa F, Suzuki Y (2007). Infectious mononucleosis-like syndrome induced by salazosulfapyridine in a patient with rheumatoid arthritis. Mod. Rheumatol..

[20] Teshima D (2003). Sulphasalazine-induced leucopenia in a patient with renal dysfunction. J. Clin. Pharm. Ther..

[21] Ohtani T, Hiroi A, Sakurane M, Furukawa F (2003). Slow acetylator genotypes as a possible risk factor for infectious mononucleosis-like syndrome induced by salazosulfapyridine. Br. J. Dermatol..

[22] Gunnarsson I (1997). Predisposing factors in sulphasalazine-induced systemic lupus erythematosus. Br. J. Rheumatol..

[23] Wadelius M, Stjernberg E, Wiholm BE, Rane A (2000). Polymorphisms of NAT2 in relation to sulphasalazine-induced agranulocytosis. Pharmacogenetics..

[24] Sabbagh N (1997). NAT2 genotyping and efficacy of sulfasalazine in patients with chronic discoid lupus erythematosus. Pharmacogenetics..

[25] Ricart E (2002). N-acetyltransferase 1 and 2 genotypes do not predict response or toxicity to treatment with mesalamine and sulfasalazine in patients with ulcerative colitis. Am. J. Gastroenterol..

[26] Tanaka E (2002). Adverse effects of sulfasalazine in patients with rheumatoid arthritis are associated with diplotype configuration at the N-acetyltransferase 2 gene. J. Rheumatol..

[27] Tanigawara Y (2002). N-acetyltransferase 2 genotype-related sulfapyridine acetylation and its adverse events. Biol. Pharm. Bull..

[28] Kumagai S (2004). N-acetyltransferase 2 genotype-related efficacy of sulfasalazine in patients with rheumatoid arthritis. Pharm. Res..

[29] Chen M (2007). N-acetyltransferase 2 slow acetylator genotype associated with adverse effects of sulphasalazine in the treatment of inflammatory bowel disease. Can. J. Gastroenterol..

[30] Taniguchi A (2007). Validation of the associations between single nucleotide polymorphisms or haplotypes and responses to disease-modifying antirheumatic drugs in patients with rheumatoid arthritis: a proposal for prospective pharmacogenomic study in clinical practice. Pharmacogenet Genomics..

[31] Hou ZD, Xiao ZY, Gong Y, Zhang YP, Zeng QY (2014). Arylamine N-acetyltransferase polymorphisms in Han Chinese patients with ankylosing spondylitis and their correlation to the adverse drug reactions to sulfasalazine. BMC Pharmacol. Toxicol..

[32] Wiese MD (2014). Pharmacogenomics of NAT2 and ABCG2 influence the toxicity and efficacy of sulphasalazine containing DMARD regimens in early rheumatoid arthritis. Pharmacogenomics J..

[33] Cascorbi I (1995). Arylamine N-acetyltransferase (NAT2) mutations and their allelic linkage in unrelated Caucasian individuals: correlation with phenotypic activity. Am. J. Hum. Genet..

[34] Wang PY, Xie SY, Hao Q, Zhang C, Jiang BF (2012). NAT2 polymorphisms and susceptibility to anti-tuberculosis drug-induced liver injury: a meta-analysis. Int. J. Tuberc. Lung Dis..

[35] Du H (2013). Slow N-acetyltransferase 2 genotype contributes to anti-tuberculosis drug-induced hepatotoxicity: a meta-analysis. Mol. Biol. Rep..

[36] Shi J, Xie M, Wang J, Xu Y, Liu X (2015). Susceptibility of N-acetyltransferase 2 slow acetylators to antituberculosis drug-induced liver injury: a meta-analysis. Pharmacogenomics..

[37] Zhang M (2018). The association between the NAT2 genetic polymorphisms and risk of DILI during anti-TB treatment: a systematic review and meta-analysis. Br. J. Clin. Pharmacol..

[38] Spinasse LB, Santos AR, Suffys PN, Muxfeldt ES, Salles GF (2014). Different phenotypes of the NAT2 gene influences hydralazine antihypertensive response in patients with resistant hypertension. Pharmacogenomics..

[39] Haroldsen PE (2015). Genetic variation in aryl N-acetyltransferase results in significant differences in the pharmacokinetic and safety profiles of amifampridine (3,4-diaminopyridine) phosphate. Pharmacol. Res. Perspect..

[40] Soejima M (2007). Association of the diplotype configuration at the N-acetyltransferase 2 gene with adverse events with co-trimoxazole in Japanese patients with systemic lupus erythematosus. Arthritis Res. Ther..

[41] Cho JH, Feldman M (2015). Heterogeneity of autoimmune diseases: pathophysiologic insights from genetics and implications for new therapies. Nat. Med..

[42] Cassotta, M., Pistollato, F. & Battino, M. Rheumatoid arthritis research in the 21st century: Limitations of traditional models, new technologies, and opportunities for a human biology-based approach. Preprint at, 10.14573/altex.1910011 (2019). 10.14573/altex.1910011 31854453

[43] Martin TD, Chan SS, Hart AR (2015). Environmental factors in the relapse and recurrence of inflammatory bowel disease: a review of the literature. Dig. Dis. Sci..

[44] Navarro F, Hanauer SB (2003). Treatment of inflammatory bowel disease: safety and tolerability issues. Am. J. Gastroenterol..

[45] Das KM, Eastwood MA, McManus JP, Sircus W (1973). Adverse reactions during salicylazosulfapyridine therapy and the relation with drug metabolism and acetylator phenotype. N. Engl. J. Med..

[46] Ma JJ (2009). Effects of NAT2 polymorphism on SASP pharmacokinetics in Chinese population. Clin. Chim. Acta..

[47] Evans DA (1969). An improved and simplified method of detecting the acetylator phenotype. J. Med. Genet..

[48] Schröder H, Evans DA (1972). Acetylator phenotype and adverse effects of sulphasalazine in healthy subjects. Gut..

[49] Khan AA, Nurazzaman M, Truelove SC (1983). The effect of the acetylator phenotype on the metabolism of sulphasalazine in man. J. Med. Genet..

[50] Mallett S, Clarke M (2002). The typical Cochrane review. How many trials? How many participants?. Int. J. Technol. Assess. Health Care..

[51] Ioannidis JP, Trikalinos TA (2007). The appropriateness of asymmetry tests for publication bias in meta-analyses: a large survey. CMAJ..

[52] Herbison P, Hay-Smith J, Gillespie WJ (2011). Meta-analyses of small numbers of trials often agree with longer-term results. J. Clin. Epidemiol..

[53] Stang A (2010). Critical evaluation of the Newcastle-Ottawa scale for the assessment of the quality of nonrandomized studies in meta-analyses. Eur. J. Epidemiol..

[54] Mantel N, Haenszel W (1959). Statistical aspects of the analysis of data from retrospective studies of disease. J. Natl Cancer Inst..

[55] DerSimonian R, Laird N (1986). Meta-analysis in clinical trials. Control. Clin. Trials..

[56] Galbraith RF (1988). A note on graphical presentation of estimated odds ratios from several clinical trials. Stat. Med..

[57] Moher D (2009). Preferred reporting items for systematic reviews and meta-analyses: the PRISMA statement. PLoS Med..

