# Supplementary material for: The association between NAT2 acetylator status and adverse drug reactions of sulfasalazine: a systematic review and meta-analysis
Source: Sci Rep. 2020 Feb 27;10:3658. doi: 10.1038/s41598-020-60467-8 (PMC7046788; doi:10.1038/s41598-020-60467-8)

**The association between *NAT2* acetylator status and adverse drug reactions of sulfasalazine: a systematic review and meta-analysis**

Jeong Yee^1*^, So Min Kim^2*^, Ji Min Han^2,3^, Nari Lee^3^, Ha Young Yoon^3^, Hye Sun Gwak^2,3†^

^1^Division of Life and Pharmaceutical Sciences, Ewha Womans University, 52 Ewhayeodae-gil, Seodaemun-gu, Seoul 03760, Republic of Korea

^2^College of Pharmacy, Ewha Womans University, 52 Ewhayeodae-gil, Seodaemun-gu, Seoul 03760, Republic of Korea

^3^Graduate School of Pharmaceutical Sciences, Ewha Womans University, 52 Ewhayeodae-gil, Seodaemun-gu, Seoul 03760, Republic of Korea

^*^The authors equally contributed to this paper.

Corresponding author: Hye Sun Gwak

College of Pharmacy and Graduate School of Pharmaceutical Sciences, Ewha Womans University, 52 Ewhayeodae-gil, Seodaemun-gu, Seoul 03760, Republic of Korea

Tel: +82-2-3277-4376; Fax: +82-2-3277-3051; E-Mail: hsgwak@ewha.ac.kr

**Supplementary Materials**

Supplementary Table S1. Sensitivity analysis of the association between *NAT2* acetylator status and overall adverse reactions of sulfasalazine by sequentially excluding each study (slow acetylators vs fast and intermediate acetylators).

Supplementary Figure S1. Galbraith plot of the association between *NAT2* acetylator status and overall adverse drug reactions of sulfasalazine.

Supplementary Table S1. Sensitivity analysis of the association between *NAT2* acetylator status and overall adverse reactions of sulfasalazine by sequentially excluding each study (slow acetylators vs fast and intermediate acetylators).

| Study excluded | Heterogeneity *I*^2^ (%) | Statistical model | Odds ratio (95% CI) |
| --- | --- | --- | --- |
| None | 64 | Random | 3.37 (1.43-7.93) |
| Sabbagh *et al*. (1997) | 67 | Random | 3.05 (1.26-7.37) |
| Ricart *et al*. (2002) | 31 | Fixed | 3.95 (2.38-6.55) |
| Tanaka *et al*. (2002) | 54 | Random | 2.55 (1.14-5.67) |
| Tanigawara *et al*. (2002) | 69 | Random | 3.30 (1.33-8.17) |
| Kumagai *et al*. (2004) | 66 | Random | 4.05 (1.61-10.18) |
| Chen *et al*. (2007) | 68 | Random | 3.28 (1.22-8.82) |
| Taniguchi *et al*. (2007) | 66 | Random | 3.11 (1.16-8.38) |
| Hou *et al*. (2014) | 69 | Random | 3.60 (1.21-10.73) |

Supplementary Figure S1. Galbraith plot of the association between *NAT2* acetylator status and overall adverse drug reactions of sulfasalazine.


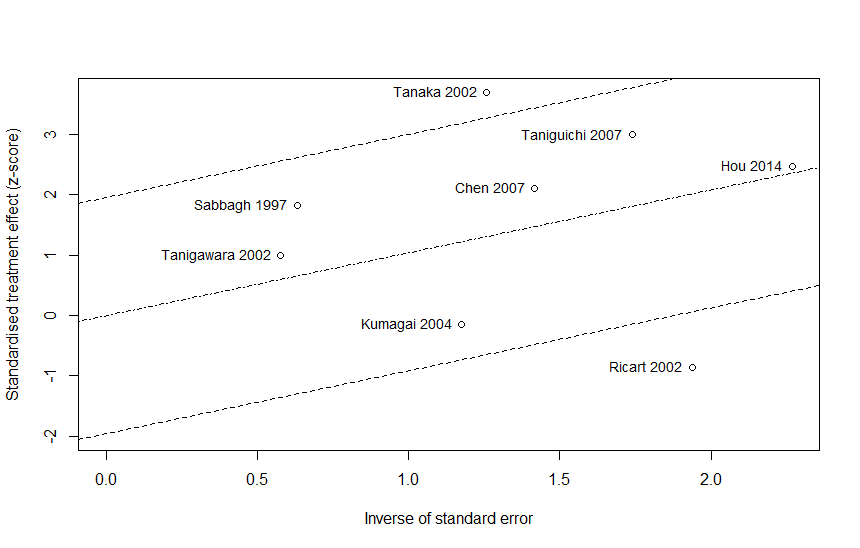

Supplement: Supplementary file 1 — Supplementary Information. [file 41598_2020_60467_MOESM1_ESM.docx]
